# The Effect of Anesthesia Type on the Stability of the Surgical View on the Monitor in Retrograde Intrarenal Surgery for Renal Stone: A Prospective Observational Trial

**DOI:** 10.3390/medicina60091435

**Published:** 2024-09-02

**Authors:** Dongwook Won, Sung Yong Cho, Hyun-Joung No, Jiwon Lee, Jin-Young Hwang, Tae Kyong Kim, Jee-Eun Chang, Hyerim Kim, Jae-Hyun Choi, Jung-Man Lee

**Affiliations:** 1 Department of Anesthesiology and Pain Medicine, Seoul National University College of Medicine, SMG-SNU Boramae Medical Center, Seoul 07061, Republic of Korea; won2501@naver.com (D.W.); jy.hwang@snu.ac.kr (J.-Y.H.); tk99@snu.ac.kr (T.K.K.); sw411528@hanmail.net (J.-E.C.); blue6935@snu.ac.kr (H.K.); 2 Department of Urology, Seoul National University College of Medicine, Seoul National University Hospital, Seoul 03080, Republic of Korea; 3 Department of Anesthesiology and Pain Medicine, Anesthesia and Pain Research Institute, Yonsei University College of Medicine, Severance Hospital, Seoul 03722, Republic of Korea; ane.nohj0410@gmail.com; 4 Department of Anesthesiology and Pain Medicine, Anesthesia and Pain Research Institute, Yonsei University College of Medicine, Gangnam Severance Hospital, Seoul 06273, Republic of Korea; belief705@yuhs.ac; 5 Department of Anesthesiology and Pain Medicine, Seoul National University Hospital, Seoul 03080, Republic of Korea; jaehyun.choi.1996@gmail.com

**Keywords:** anesthesia, nephrolithiasis, renal stone, retrograde intrarenal surgery

## Abstract

*Background and Objectives*: Retrograde intrarenal surgery (RIRS) is a minimally invasive technique for nephrolithiasis. RIRS is performed via a monitor screen displaying a magnified surgical site. Respiration can affect the stability of the surgical view during RIRS because the kidneys are close to the diaphragm. The purpose of this trial is to compare the effect of anesthesia type on the stability of the surgical view during RIRS between spinal anesthesia and general anesthesia. *Materials and Methods*: Patients were allocated to the general anesthesia group or spinal anesthesia group. During surgery, movement of the surgical field displayed on the monitor screen was graded by the first assistant on a 10-grade numeric rating scale (0–10). Next, it was also graded by the main surgeon. After surgery, we evaluated the discomfort with the anesthesia method for all patients. *Results*: Thirty-four patients were allocated to the general anesthesia group and 32 patients to the spinal anesthesia group. The average values of the two surgeons for surgical field oscillation grade showed vision on the monitor screen was more stable in the general anesthesia group than the spinal anesthesia group (3.3 ± 1.6 vs. 5.0 ± 1.6, *p* < 0.001). The degrees of the inconvenience of the surgery did not differ between the groups (0.7 ± 1.8 vs. 1.6 ± 2.6, *p* = 0.114), even though more patients reported inconvenience with a grade of 3 or more in the spinal anesthesia group (8.8% vs. 28.1%, *p* = 0.042). *Conclusions*: In terms of the visualization of the surgical site, general anesthesia might provide a more stable surgical view during RIRS compared to spinal anesthesia without increasing inconvenience induced by the type of anesthesia.

## 1. Introduction

Retrograde intrarenal surgery (RIRS) has been widely used for the treatment of renal stones resistant to initial extracorporeal shock wave therapy (ESWT), and unfavorable anatomy for ESWT such as steep infundibular-pelvic angle, long low lower pole calyx, or narrow infundibulum. With the advancement of the flexible ureteroscope, the indications for the RIRS have been expanded, including large stones > 20 mm, ureteral stricture, and urothelial carcinoma [1].

During RIRS, surgeons break the renal stone into small pieces by lasing it while they see the surgical field on a video screen. During the process, the laser beam can injure the calyceal mucosa if the stone gets out of the target because it can roll in the space by the shock of the laser. At the same time, the view of the scope cannot be fixed to a specific location of the calyx because the kidney moves as the patient breathes [2]. As the flexible ureterorenosope tip approaches the calyx closely, a small movement of the kidney might considerably influence the stationariness of the view. Meanwhile, with more movement of the surgical field on the monitor screen, the surgeon can feel more fatigued and might not concentrate as time passes. Consequently, the instability of the surgical view may decrease the success rate of the procedure, prolong operative time, and increase complications. Therefore, any technique that can minimize the surgical field movement or enhance the regularity of movement on the video screen might help to provide comfortable operating conditions to surgeons and reduce mucosal injury during RIRS. RIRS is usually performed under general anesthesia with mechanical ventilation or regional anesthesia, in which the patient’s lungs are ventilated by self-respiration [3]. A previous study suggested a lung ventilation strategy with periodic apnea to overcome stone movement by the patient’s lung ventilation [4]. Recently, studies about the effect of respiration on the condition of RIRS or ESWT for renal stones have been introduced [5,6]. However, there has been no study comparing the effects of different anesthesia types on the condition of RIRS for renal stones in terms of the stability of the surgical field on the monitor screen.

The purpose of the study was to compare the effect of anesthesia type on the stability of the surgical view during RIRS between spinal anesthesia and general anesthesia.

## 2. Materials and Methods

This prospective study was approved by the institutional review board of the Seoul Metropolitan Government Seoul National University Boramae Medical Center (20-2017-10) and was registered at ClinicalTrials.gov (initial release, 11 December 2017, NCT03373617) before enrollment of subjects. Written informed consent was obtained from all subjects before the study. Adult patients (≥18 years old) with an American Society of Anesthesiologists (ASA) physical status classification of I-III who were scheduled for an elective RIRS to remove renal stones larger than 10 mm under general anesthesia or spinal anesthesia were recruited from 13 December 2017 to 14 December 2018. Patients with a history of lung surgery, pulmonary disease, such as acute respiratory distress syndrome, chronic obstructive pulmonary disease, pneumonia, or contraindication of spinal anesthesia were excluded from this study.

After enrollment, each patient was assigned to one of two groups according to the patient’s wishes for the type of anesthesia, which was general anesthesia (G group) or spinal anesthesia (S group). The medical team provided enough information on both types of anesthesia but did not influence the patients in choosing the anesthesia type. However, we explained the possibility of changing the anesthesia type from spinal anesthesia to general anesthesia due to failure of spinal anesthesia for any reason at the pre-anesthesia visit before the surgery. For each patient, their sex, age, height, and weight were recorded.

Patients were admitted into the operating room without premedication. Electrocardiography, non-invasive arterial blood pressure, and peripheral oxygen saturation monitoring started. For the G group, 1% lidocaine 30 mg, propofol 1.5 mg/kg, and fentanyl 50–100 μg were administered for induction of anesthesia. Next, rocuronium 0.6 mg/kg was administered for neuromuscular blockade. An anesthesiologist performed tracheal intubation, and the patient’s lungs were ventilated with a tidal volume of 8 mL/kg and a positive end-expiratory pressure of 5 cmH_2_O. The respiratory rate of mechanical ventilation was adjusted to maintain the end-tidal carbon dioxide between 30 and 35 cmH_2_O.

For the S group, the patient was positioned laterally in the fetal position. Sterile preparation with a solution of 2% chlorhexidine gluconate and 72% ethanol was performed around the L2-S1 skin area of the back. Spinal anesthesia was performed with a 25-gauge spinal needle with Quincke bevel (TaeChang Industrial Co., Gongju, Republic of Korea) by a standing anesthesiologist at the level of the L3–4 or L4–5 interspinous process space as the insertion site. After confirmation of clear cerebrospinal fluid leakage by successful dural puncture, 0.5% hyperbaric bupivacaine with fentanyl 20 μg was injected. The dose of bupivacaine was determined by the standing anesthesiologist to target the anesthesia level up to approximately the T6 skin segment. If spinal anesthesia failed with up to three attempts, the patient would be scheduled to undergo general anesthesia as previously explained.

**Figure 1:**
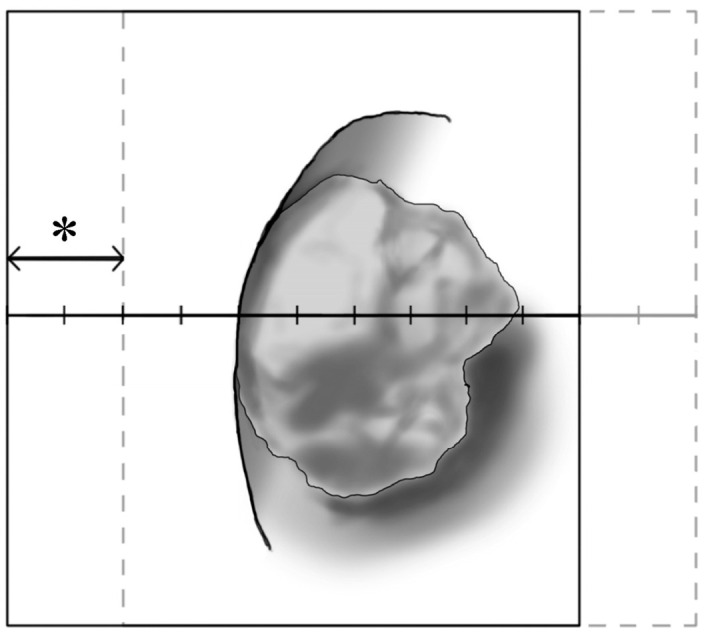
Diagram demonstrating grading of screen movement. In this case, screen movement (*) is graded as 2, which means that the surgical field of view on the monitor screen oscillates by 20% of the total screen size.

After the establishment of adequate anesthesia with one of both maneuvers, surgical preparation was performed by a surgical assistant, who was not involved in the surgical procedure. To blind the surgeons to the type of anesthesia, the anesthesia machine and anesthesia faculty were screened from the surgical field with a sterile drape. Thereafter, surgeons, including a main surgeon (S.Y.C.) and a surgical assistant, entered the operating theater and inserted a flexible ureteroenoscope into the target site of surgery through the urethra, bladder, and ureter. When the vision of the surgical field was obtained on the monitor screen, an investigator asked the first assistant, who was a resident with experience of 2–3 years in the department of urology, to grade how much the vision oscillated with a 10-grade numeric rating scale (Grade 1) (0: no movement, 10: movement about the size of the entire monitor screen). That is, the resident was first asked to divide the monitor screen into ten equal parts. The resident was then asked to estimate the extent to which specific structures, such as renal stone or calyx, that appeared on the monitor screen shook on the screen. If such structures on the monitor screen shook to an extent equivalent to about 20% of the entire monitor screen, the resident was asked to rate the degree of oscillation of the vision as a ‘2’ (Figure 1). Then, the investigator asked the main surgeon (S.Y.C.), who was not aware of the score that the assistant had given, to grade how much the vision moved with the same scale (Grade 2). Finally, we defined the average grade (AG) of Grade 1 and Grade 2 in each patient as a primary outcome. We summed the duration of laser irradiation for each patient during surgery.

After surgery, the occurrence of high fever (≥38 °C) was monitored over 7 postoperative days for all patients. Additionally, we called patients to ask how much they did grade inconvenience caused by anesthesia with a scale of 0 to 10 (0: no inconvenience, 10: maximum inconvenience) after discharge to investigate all kinds of inconvenience caused by the type of anesthesia that they received for the surgery.

The stone type was confirmed by Fourier-transform infrared spectroscopy for stone analysis. The location and size of the stone were confirmed by evaluating the computed tomography images performed before surgery. The total stone volume was calculated by the number of stones along with a generic formula of ‘length × breadth × height × 0.523’, assuming that the stones were ellipsoid.

All continuous variables are expressed as mean ± standard deviation (SD) or as the median [interquartile range]. For the grades of vision oscillation for the surgical field, which were evaluated by the assistant residents (Grade 1) and the main surgeon (Grade 2), we analyzed the agreement degree between them and their average grade (AG) using an intraclass correlation coefficient with a two-way random model. Then, we compared the AG, which was defined as a primary outcome in our study, between the two groups using Student’s t-test. Lasing time during surgery was compared between the two groups using the Mann–Whitney U test.

Categorical variables were expressed in numbers (%) and tested with the χ^2^ test or Fischer’s exact test. We compared the degree of inconvenience that patients felt using Fischer’s exact test. A *p*-value less than 0.05 was considered statistically significant. Statistical analyses were performed with SPSS Statistics 26.0 software (IBM Corporation, Chicago, IL, USA).

## 3. Results

A total of 66 patients who underwent elective RIRS were enrolled between December 2017 and December 2018 in the present study. There was no harmful event for any patient in the trial. 

Thirty-four patients were allocated to the G group and 32 patients to the S group. For the 32 patients of the S group, the dose of bupivacaine injected intrathecally was 11–16 mg (median: 14 mg). Among them, the insertion site of the spinal needle was the L4–5 interspinous process space for 29 patients and the L3–4 interspinous process space for 3 patients. In one patient of the S group, anesthesia was changed to general anesthesia after evaluation of the surgical field movement on the screen monitor because the patient complained of pain during the examination. The patient was a 41-year-old man with a height of 171.5 cm and a weight of 79.4 kg. The insertion site of the spinal needle was the L4–5 interspinous process space, and the dose of 0.5% hyperbaric bupivacaine injected intrathecally was 13 mg. The block height of the patient was T10 of the skin dermatome. For the other 31 patients with successful spinal anesthesia in the S group, block heights were T8 of skin dermatome or upper, up to C7 for one patient for whom bupivacaine 16 mg was administered intrathecally. No patient had difficulty in self-respiration due to the high level of spinal anesthesia.

The patients’ characteristics were not significantly different between the two groups (Table 1). In addition, no patient had a congenital anomaly, such as ureteropelvic junction obstruction in the kidney. Table 2 shows the stone position and the types and times of the procedures, including lasing time, operation time, and anesthesia time of the two groups in this trial.

The means ± SD of the surgical field oscillation grades for the G group and S group evaluated by the assistant (Grade 1) were 3.3 ± 1.5 and 5.2 ± 1.7, respectively. Those values evaluated by the main surgeon (Grade 2) were 3.3 ± 1.8 and 4.8 ± 1.7, respectively (Table 3). The agreement of grading between the two surgeons was substantially reliable with an intraclass correlation coefficient for the average value of 0.889 (95% confidence interval (CI); 0.819–0.932). The average grade (AG) of the two surgeons for surgical field oscillation showed that vision on the monitor screen was more stable in the G group than the S group (3.3 ± 1.6 vs. 5.0 ± 1.6, mean difference 1.7 ± 0.4, 95% CI 1.0 to 2.5, *p* < 0.001).

When we followed up with the patients for any inconvenience associated with the anesthesia type, patients with a score of 3 or higher for inconvenience were less numerous in the G group than the S group (8.8% vs. 28.1%, *p* = 0.042). Three patients who provided a score of 3 or higher in the G group complained of sore throat or nausea. Nine patients who answered a score of 3 or higher in the S group complained of headache, pain on the needle insertion site during the procedure of spinal anesthesia, or pain at the operation site because of incomplete spinal anesthesia, which was a low level of spinal anesthesia (T 10). However, there was no difference regarding the absolute value of degrees for inconvenience between the G group and the S group (*p* = 0.216).

There was no difference in fever occurrence during the postoperative 7 days between the G group and the S group (14.7% vs. 9.4%, *p* = 0.710).

## 4. Discussion

We showed that general anesthesia could provide better surgical conditions with more stable visualization of the surgical field during RIRS compared to spinal anesthesia. Additionally, we found that more patients felt inconvenience, which was grade 3 or more on a 0–10 scale, with spinal anesthesia than with general anesthesia in our study population, even though the difference in the absolute score itself for inconvenience was not significant between general anesthesia and spinal anesthesia.

A previous study suggested a lung ventilation strategy with periodic apnea to prevent stone movement by the patient’s lung ventilation [4]. However, the technique of using periodic apnea should be unstable in terms of concern about hypercapnia, hypoxia, and discontinuity in the fragmentation of the renal stones. A recent study presented that slow respiration showed good efficiency with higher hit rates for renal stones and a low incidence of cavitation damage during lithotripsy in vitro. In addition, the previous study showed that the more regular respiration was, the higher the stone fragmentation efficiency (66.7% vs. 36.8%) was in vivo when all 52 participants who received ESWT were awake [6]. Another previous study presented that high-frequency jet ventilation with small volume mechanical ventilation could reduce respiratory kidney motion, leading to better conditions for RIRS [5]. In addition, a previous study showed that a ventilation mode using a combined strategy with a high frequency and a relatively small tidal volume could provide the surgeon with the ability to target the laser more effectively during RIRS for renal stone under general anesthesia [7]. These previous studies did not compare the effects of the different anesthesia types, such as ‘general anesthesia vs. spinal anesthesia’ on the conditions of RIRS. However, the results of our study seemed to be consistent, even though indirectly, with the results of those previous studies, in terms of the advantage of regular patient lung ventilation with a small tidal volume for the stable vision of the surgical field during RIRS. One previous study showed general anesthesia provided the surgeon more comfort in laser focusing during surgery than spinal anesthesia (7.6 vs. 6.3), by scoring between 1 (very poor) and 10 (very good) [8]. It seems that our research results should provide a fundamental reason consistent with that finding of the previous study. However, we did not directly investigate the effect of the stability of the surgical field on the monitor screen on the operation as well as the postoperative complication rate, stone free rate, and the incidence of re-operation in the study.

Surgical procedures can become more traumatic to patients unless meticulous concentration and ideal surgical conditions are maintained. Any unexpected or superfluous movement in the surgical field might exert a harmful effect on the patient during surgery. Moreover, the deep neuromuscular block could be helpful for surgical conditions of various surgeries [9,10,11,12,13,14].

RIRS is an established alternative to ESWL or PCNL in patients with low-volume stones, especially for ESWL-resistant stones and for patients with co-morbidities [15]. It is a minimally invasive video-assisted technique. The surgical view through a long and small diameter scope should be magnified to achieve successful treatment of renal stones using this technique, and the manipulation of the stones at the surgical site is limited. Therefore, even small movements of the stone might hinder the surgical process or harm the patient. RIRS is performed while patients are breathing spontaneously or mechanically. As the patient’s lungs are ventilated, the vision on the monitor screen through the ureterorenoscope inserted into the renal tract can inevitably move. The manner of lung ventilation, which is spontaneous or mechanical, might affect the intraoperative stability of the surgical view during RIRS, as anesthesia and neuromuscular blockade affect the movement of the diaphragm [9], and mechanical ventilation with the aid of neuromuscular blockade must be more regular than spontaneous ventilation. In a study, when a person breathed spontaneously, the dorsal part of the diaphragm moved preferentially. On the contrary, when the patient was paralyzed, the non-dependent part of the diaphragm moved more, that is, the ventral part, when the patient was in a supine position [16]. Additionally, the contribution of diaphragm movement to tidal volume decreases when the patient is anesthetized and paralyzed [17]. In a study by Krayer et al., the contribution of the diaphragm to tidal volume was nearly 100% when patients were awake. On the contrary, the contribution decreased to half of the tidal volume when patients were paralyzed [17]. As the kidneys are in the retroperitoneal space, the movement of the dorsal rather than the ventral diaphragm might induce the excessive movement of kidneys, resulting in the instability of the surgical view through the ureterorenoscope. Therefore, the reduced contribution of the diaphragm to the tidal volume might decrease surgical field movement on the monitor screen during RIRS. Additionally, breathing in awake patients can be affected by the psychological mood of the patients, and irregular breathing can occur in dozing or sedated patients during surgery, which might lead to irregular diaphragm movement. In our study, surgeons reported that the view was more stable when the patient was paralyzed under general anesthesia than during spinal anesthesia. RIRS is a minimally invasive treatment option for renal stones untreated by ESWL. Meanwhile, it requires the surgeon’s steady concentration on the surgical field, imposing stress and strain on the surgeon. The stabilization of the surgical field on the monitor screen might reduce the time needed to search for and aim at the stones and the stress of surgeons, resulting in improved surgical outcomes. However, these surgical outcomes were not evaluated in our study.

When we compared the discomfort about anesthesia that the patient was provided with for the surgery, there was no significant difference between the two groups, even though spinal anesthesia can be helpful for pain relief in the first several hours up to 24 h of the postoperative period [18,19,20,21,22].

RIRS is usually performed while the patient is in a lithotomy position, which enables surgeons to visualize and access the perineal region of the patient. However, the position might cause great discomfort and a sense of vulnerability and humiliation to patients when they are awake, which might make the surgical experience unsatisfactory [23]. Feeling embarrassment and defenselessness might make the same illness more painful for the patient [24]. In a previous study, the patient’s fear of the other technique was the second main factor influencing the choice of anesthesia type [25]. In our study, three patients who answered a score of 3 or higher in the G group complained of sore throat or nausea. We used tracheal tubes in all patients who received general anesthesia in the study. However, supraglottic airway devices can be useful in most patients for RIRS, and those can alleviate sore throats [26]. Additionally, antiemetics can prevent or alleviate postoperative nausea [27].

In our results, the operation time and anesthesia time were longer in the G group than in the S group. Typically, general anesthesia takes longer than spinal anesthesia because of the time it takes to recover consciousness and muscle strength from general anesthesia. Meanwhile, some previous studies have shown that the operation time in surgeries with ureteroscopy is affected by the specific stone position, the intraoperative use of the ureteric access sheaths, the pre-operative use of stents, or the application of post-operative stenting [28,29,30,31]. Additionally, we think that the methods for stone removal, namely capture in the basket following fragmentation or dusting and suction, could affect the operation time in the present study. However, our study did not investigate factors that may affect surgical time.

There were some limitations in our study. First, we could not conduct this trial with randomization in assignment to a group in all patients. We asked patients the type of anesthesia they preferred. According to their answer, we assigned the patient to one of the two groups (the G group or the S group) according to their preference, because the anesthesia type should be applied according to the patient’s wishes unless there is a medical or surgical limitation. Therefore, this trial had the weakness of not being a randomized controlled trial. However, we performed this study while blinding the main surgeon and the assistant who evaluated the primary outcome. Additionally, there was no significant difference in the characteristics of the patients between the two groups. Allowing patients to choose the anesthesia type might lead to a bias on the inconvenience. Patients’ preferences for a certain anesthesia method (GA vs. SA) may be due to their aversion to other anesthesia methods because of their negative preconceptions about them. We think that the bias might have been exacerbated if the patients had been asked about their discomfort after experiencing the anesthesia method, which they disliked, through a complete random assignment. Second, the ratings of surgical field oscillation obtained from the first assistant resident and the main surgeon were somewhat subjective and therefore not validated. In order to validate those ratings more objectively, there may be a method to draw nine straight lines horizontally and vertically on the monitor screen for them to more objectively view the degree of oscillation of the surgical field on the monitor screen. However, considering that the grade scores for each group evaluated by the first assistant resident and the main surgeon were similar (3.3 ± 1.5 and 3.3 ± 1.8 in the G group, 5.2 ± 1.7 and 4.8 ± 1.7 in the S group), we believe that the method used to evaluate the degree of surgical field oscillation in this study was unlikely to have undermined the validation. Third, we compared the patient’s inconvenience with the numeric rating scale (0–10) after the surgery. However, interpreting the results was limited because the score was not validated. We asked patients about any inconvenience related to anesthesia type in open questions and made them grade their experience like on a numeric rating scale for pain. However, inconvenience is an ambiguous emotional reaction to events. The Iowa Satisfaction with Anesthesia Scale categorized patients’ experience with monitored anesthesia care into 11 statements such as nausea, pain, or itching, and this makes the evaluation more objective [32]. Additionally, we could not compare patients’ experiences with general and spinal anesthesia with the same grading system because inconvenience with general anesthesia should have a different etiology compared with spinal anesthesia. We did not investigate specific kinds of inconvenience because general anesthesia and spinal anesthesia have different characteristics in terms of postoperative complications and complaints. Moreover, we could not obtain complete information from each patient to compare the inconvenience between general anesthesia and spinal anesthesia because they did not experience both types of anesthesia simultaneously during this trial. Therefore, interpretation of our result about inconvenience from the type of type should be performed with caution.

## 5. Conclusions

In terms of the visualization of the surgical field on the monitor screen through the ureterorenoscope, general anesthesia could provide a more stable surgical view during RIRS compared to spinal anesthesia without increasing the inconvenience induced by the type of anesthesia.

## Figures and Tables

**Table 1:** Patient characteristics.

Patient Characteristics	G Group	S Group	*p*-Value
	(*n* = 34)	(*n* = 32)	
Sex (M/F)	18/16	21/11	0.295
Age (y)	55.6 ± 15.7	51.5 ± 13.9	0.268
Height (cm)	162.2 ± 10.9	163.3 ± 9.8	0.668
Weight (kg)	64.8 ± 15.0	67.4 ± 12.2	0.433
BMI (kg/m^2^)	24.4 ± 3.8	25.3 ± 4.0	0.380
ASA physical status class (I/II/III)	16/13/5	19/12/1	0.234

BMI, body mass index; ASA, American Society of Anesthesiologists. Data are presented as mean ± standard deviation or numbers.

**Table 2:** Operation characteristics.

Operation Characteristics	G Group	S Group	*p*-Value
	(*n* = 34)	(*n* = 32)	
Dominant stone type	6/22/1/3/0	2/26/0/0/3	0.054
(UA/COM/COD/St/CA)			
Stone position	17/8/7	18/8/6	0.949
(LK/RK/BK)			
Number of stones	2 [1, 3]	2 [1, 3]	0.649
Total stone volume (cm^3^)	0.5 [0.2, 0.9]	0.4 [0.2, 0.8]	0.496
Lasing time (min)	21 [10, 35]	20 [5, 30]	0.418
Operation time (min)	62 [45, 90]	50 [39, 67]	0.048
Anesthesia time (min)	101 ± 36	82 ± 25	0.014

UA, uric acid; COM, calcium oxalate monohydrate; COD, calcium oxalate dihydrate; St, struvite; CA, carbonate apatite; LK, left kidney; RK, right kidney; BK, both kidneys. Data are presented as means ± standard deviation, medians [interquartile range], or numbers.

**Table 3:** Difference in surgical field oscillation on the monitor screen between the two groups.

Surgical Field Oscillation	G Group	S Group	MD (95% CI)	*p*-Value
	(*n* = 34)	(*n* = 32)		
Grade 1	3.3 ± 1.5	5.2 ± 1.7	1.9 ± 0.4 (1.1, 2.7)	<0.001
Grade 2	3.3 ± 1.8	4.8 ± 1.7	1.5 ± 0.4 (0.7, 2.4)	<0.001
AG	3.3 ± 1.6	5.0 ± 1.6	1.7 ± 0.4 (1.0, 2.5)	<0.001

The grade was from 0 to 10 (0, no oscillation, 10, maximum oscillation— full monitor size or more). Grade 1; grade of surgical field oscillation which was scored by the assistant, Grade 2; grade of surgical field oscillation which was scored by the main surgeon, AG; average grade of Grade 1 and Grade 2, MD; mean difference, CI; confidence interval. Data are presented as means ± standard deviation.

## Data Availability

The datasets generated for this study are available on request to the corresponding author.
